# Improving Osteogenesis Activity on BMP-2-Immobilized PCL Fibers Modified by the *γ*-Ray Irradiation Technique

**DOI:** 10.1155/2015/302820

**Published:** 2015-05-18

**Authors:** Young-Pil Yun, Jae Yong Lee, Won Jae Jeong, Kyeongsoon Park, Hak-Jun Kim, Jae-Jun Song, Sung Eun Kim, Hae-Ryong Song

**Affiliations:** ^1^Department of Orthopedic Surgery and Rare Diseases Institute, Korea University Medical College, Guro Hospital, No. 80, Guro-dong, Guro-gu, Seoul 152-703, Republic of Korea; ^2^Department of Biomedical Science, College of Medicine, Korea University, Anam-dong, Seongbuk-gu 136-701, Republic of Korea; ^3^Department of Molecular & Cell Biology, University of California, 142 LSA No. 3200, Berkeley, CA 94720-3200, USA; ^4^Division of Bio-Imaging Chuncheon Center, Korea Basic Science Institute, 192-1 Hyoja 2-dong, Chuncheon, Gangwon-do 200-701, Republic of Korea; ^5^Department of Otorhinolaryngology-Head and Neck Surgery, Korea University Medical College, Guro Hospital, No. 80, Guro-dong, Guro-gu, Seoul 152-703, Republic of Korea

## Abstract

The purpose of this study was to demonstrate the ability of BMP-2-immobilized polycaprolactone (PCL) fibers modified using the *γ*-ray irradiation technique to induce the osteogenic differentiation of MG-63 cells. Poly acrylic acid (AAc) was grafted onto the PCL fibers by the *γ*-ray irradiation technique. BMP-2 was then subsequently immobilized onto the AAc-PCL fibers (BMP-2/AAc-PCL). PCL and surface-modified PCL fibers was characterized by evaluation with a scanning electron microscope (SEM), X-ray photoelectron spectroscopy (XPS), and contact angle. The biological activity of the PCL and surface-modified PCL fibers were characterized by alkaline phosphatase (ALP) activity, calcium deposition, and the mRNA expression of osteocalcin and osteopontin in MG-63 cells. Successfully grafted AAc and PCL fibers with immobilized BMP-2 were confirmed by XPS results. The results of the contact angle showed that BMP-2/AAc-PCL fibers have more hydrophilic properties in comparison to PCL fibers. The ALP activity, calcium deposition, and gene expressions of MG-63 cells grown on BMP-2/AAc-PCL fibers showed greatly induced osteogenic differentiation in comparison to the PCL fibers. In conclusion, these results demonstrated that BMP-2/AAc-PCL fibers have the potential to effectively induce the osteogenic differentiation of MG-63 cells.

## 1. Introduction

The use of graft materials is a requisite to regenerating the bone defects caused by trauma, inflammation, disease, and fracture. Among graft materials, autologous bone grafts with osteoconductivity, osteoinductivity, and osteogenecity are widely used as the gold standard for the treatment of bone repair and regeneration. Despite these beneficial properties, major shortcomings such as their limited supply and donor-site morbidity restrict their use in clinical practice [1–3]. To address these shortcomings, many experts have recently been experimenting to replace autologous bone grafts with artificial bone graft materials such as metal, ceramics [hydroxyapatite (HAp), *β*-tricalcium phosphate (*β*-TCP), and biphasic calcium phosphate (BCP)], and collagen sponges [2, 4–6]. In addition, for effective bone repair and regeneration, the substrates need to be capable of inducing osteogenesis in combination with growth factors. Therefore, to support these conditions, the design of these substrates must be considered not only with respect to their three-dimensional porous structures, but also with respect to their mimicry of the extracellular matrix (ECM) in order to produce engineered bone tissue. In this regard, nanofibrous scaffolds offer many advantageous properties, including high surface area, macroporosity, and combined micro- and nanoscale roughness [7, 8]. Moreover, nanofibrous scaffolds may promote cell adhesion and proliferation as well as protein adsorption, osteoblasts differentiation, and biomineralization [9]. Nanofibrous scaffolds can be prepared by several methods such as thermally induced liquid-liquid phase separation, self-assembly, or carbon dioxide laser supersonic drawing and electrospinning [10–15]. Among these methods, electrospinning is one of the most widely used because of its simplicity and its ability to produce continuous fibers on a large scale. Although nanofibrous scaffolds have many benefits, they are sometimes modified with bioactive molecules using plasma treatment, etching, or *γ*-ray irradiation to improve the differentiation and mineralization of osteoblasts or mesenchymal stem cells (MSCs) [16–19].

Bone morphogenic proteins (BMPs), which play an essential role in bone and cartilage regeneration, have been widely used in bone grafts. Among the BMPs, BMP-2 has very powerful osteoinductive properties that play key roles during bone formation [20, 21]. Boyne [22], Herford and Boyne [23], and Maegawa et al. [24] have reported that BMP-2 induced the differentiation of mesenchymal stem cells (MSCs) into osteogenic cells to repair facial skeleton defects and large bone defects and fuse the spine. In published studies, BMP-2 also showed evidence of inducing the osteoblastic gene markers such as osteocalcin, osteopontin, bone sialoprotein, and alkaline phosphatase (ALP) during osteogenic differentiation* in vitro* [25, 26]. In our previous studies, MG-63 cells (osteoblast-like cells) or periodontal ligament cells (PDLCs) grown on BMP-2 immobilized onto or incorporated into titanium (Ti) or PCL fibers resulted in significantly different osteoblast differentiation in comparison to those on Ti or PCL fibers alone [27–30].

The modification of substrates using *γ*-ray irradiation provided a major advantage in that it did not require the use of catalysts or additives to initiate the reaction [31]. Moreover, the *γ*-ray irradiation technique was capable of uniformly and rapidly grafting active radical sites on the surface of polymer materials [32, 33].

This study aimed to develop PCL fibers with immobilized BMP-2 modified by the *γ*-ray irradiation technique to induce the osteogenic differentiation of MG-63 cells.

## 2. Materials and Methods

### 2.1. Materials

Polycaprolactone (PCL, Mn 70,000), 2-aminoethyl methacrylate hydrochloride, and fluorescamine were purchased from Sigma (Saint Louis, MO, USA). Methanol and tertahydrofuran (THF) were purchased from Duksan Chemical (Seoul, Korea). N,N-dimethyl formamide (DMF) was purchased from Showa Chemical (Japan).* E. coli*-derived recombinant human bone morphogenetic protein-2 (BMP-2) was donated by Cowellmedi (Busan, Korea). Dulbecco's modified eagle's medium (DMEM), fetal bovine serum (FBS), phosphate-buffered saline (PBS), and penicillin-streptomycin were purchased from Gibco-BRL (Rockville, MD, USA). The cell counting kit-8 (CCK-8) was purchased from Dojindo (Tokyo, Japan). The BMP-2 ELISA kit was purchased from PeproTech Inc. (Rocky Hill, NJ, USA). All chemicals and solvents were used without further purification. MG-63 cells (human osteosarcoma cell line) were purchased from Korea Cell Line Bank (KCLB number 21427, Seoul, Korea).

### 2.2. Preparation of the PCL Fibers

To prepare the polycaprolactone (PCL) solution, PCL was dissolved with a concentrate of 12% (w/v) in a mixture of THF and DMF (70 : 30, v/v) at room temperature (RT). The solution was then injected using an electrospinning system. The electrospinning system consisted of a high-voltage power supply (ESR-200RD, NanoNC, Korea), an infusion pump (SHB366, Sckjmotor, China), a stainless-steel blunt-ended needle (20 G, NanoNC), a 12 mL plastic syringe (Henke Sass Wolf, Germany), and a rotating collecting drum. The PCL solution was placed within a plastic syringe fitted with a stainless-steel needle. The collector was positioned at a fixed distance of 15 cm from the needle. The infusion pump was utilized to keep the feeding rate of the solution constant at 1.0 mL/hr. A fixed voltage of 11.2 kV was constantly applied between the needle and the collecting drum. The electrospun fibers were collected on a rotating collecting drum wrapped with aluminum foil with a diameter of 6 cm.

### 2.3. AAc-Grafted PCL Fiber

For the AAc grafting, the electrospun PCL fiber was immersed in different concentrations of 1 wt% AAc solution within methanol, and the samples were exposed to gamma ray irradiation (ACEL type C-1882, Korea Atomic Energy Institute). The total radiation dose was at a rate of 10 kGy/hr, and homopolymers and unreacted monomers were washed by deionized water (DW) for 4 hr after irradiation.

### 2.4. Immobilization of BMP-2 on the PCL Fiber

BMP-2 was immobilized on the surface of the AAc-PCL fiber via electrostatic interactions between the highly positive-charged BMP-2 and the highly negative-charged AAc. In brief, the AAc-PCL fiber was placed into the MES buffer (pH 5.6), and then BMP-2, at a concentration of 500 ng/mL, was added to the MES buffer (BMP-2/AAc-PCL fiber). The reaction was maintained for 6 hr. After the reaction, the BMP-2/AAc-PCL fiber was rinsed with DW and lyophilized for 3 days. In addition, BMP-2 was immobilized on the surface of the PCL (BMP-2/PCL fiber). The procedure for immobilizing BMP-2 on the PCL surface was similar to that for immobilizing the BMP-2/AAc-PCL fiber.

### 2.5. Characterization of PCL Fibers and Surface-Modified PCL Fibers

Surface morphologies of the PCL, AAc-PCL, BMP-2/PCL, and BMP-2/AAc-PCL fibers were observed using the scanning electron microscopy (SEM, S4800, Hitachi, Japan). Gold was coated onto the specimens using a sputter-coater (Eiko IB, Japan) and SEM was operated at 3 kV. X-ray photoelectron spectroscopy (XPS) on a K-alpha spectrometer (ESCALAB250 XPS System, Theta Probe AR-XPS System, Thermo Fisher Scientific, UK) with an Al K*α* X-ray source (1486, 6 eV photons) at the Korea Basic Science Institute Busan Center which was used to confirm the surface composition of the PCL, AAc-PCL, BMP-2/PCL, and BMP-2/AAc-PCL fibers. The C1s hydrocarbon peak at 284.84 eV was used as the reference for all binding energies. PCL fibers and surface-modified PCL fibers were also investigated using contact angles. Contact angles were measured with a goniometer (SEO Phoenix 300, Suwon, Korea). To investigate the amount of grafted AAc on the PCL fiber, the toluidine blue staining method was used as previously described [19]. In brief, PCL fibers grafted with AAc were immersed onto toluidine blue solution (0.01 M HCl, 20 mg NaCl, and 4 mg toluidine blue O chloride) for 4 hr at room temperature (RT). After the reaction, the substrate was rinsed with distilled water (DW). After being immersed into the toluidine blue solution, the substrate was vigorously vortexed in a 0.1 M NaOH and ethanol solution (1 : 4, v/v) to completely decolorize the stained substrate, and then the amount of toluidine blue O released from the substrate was quantified by measuring the absorbance at 630 nm using a microplate reader (Bio-Rad, Hercules, CA, USA).

### 2.6. Release Study

The release profiles of BMP-2 from the BMP-2/PCL fibers and BMP-2/AAc-PCL fibers were evaluated by an enzyme-linked immunosorbent assay (ELISA) kit according to the manufacturer's instructions. In brief, BMP-2/PCL fibers and BMP-2/AAc-PCL fibers were placed into a 50 mL conical tube (Falcon, USA) containing 1 mL PBS buffer (pH 7.4) with gentle shaking at 100 rpm and 37°C. At predesignated time intervals of 1, 3, 5, and 10 hr, and 1, 3, 5, 7, 14, 21, and 28 days, PBS was extracted and collected from the specimens and fresh PBS was replaced into the 50 mL conical tube. The amount of BMP-2 released was determined by an ELISA kit using a microplate reader at 450 nm.

### 2.7. Cell Proliferation

MG-63 cells which were isolated from human bone tissues were used because MG-63 cells are popular cells to demonstrate the osteogenic effects of drugs, peptides, or proteins in/on various substrates. MG-63 cells at a concentration of 1 × 10^5^ cells/mL were seeded onto PCL, AAc-PCL, BMP-2/PCL, and BMP-2/AAc-PCL fibers in a 24-well tissue-culture plate and maintained with DMEM supplemented with 10% FBS and 1% antibiotics. After 1, 3, and 7 days of culturing, substrates were rinsed with PBS and CCK-8 proliferation reagents were added to the substrates and incubated for 1 hr. Reagents were transferred to 96-well tissue-culture plates. Optical density was measured using a microplate reader at a wavelength of 450 nm.

### 2.8. Alkaline Phosphatase (ALP) Activity

ALP activity was measured to evaluate the early osteogenic differentiation of MG-63 cells onto PCL fibers and surface-modified PCL fibers after 3, 7, and 10 days of incubation. Briefly, MG-63 cells were seeded at a density of 1 × 10^5^ cells/mL onto the surface of PCL fibers and surface-modified PCL fibers into a 24-well tissue-culture plate and maintained in osteogenic medium (DMEM supplemented with 10% FBS, 50 *μ*g/mL ascorbic acid, 10 nM dexamethasone, and 10 mM *β*-glycerophosphate in the presence of 100 U/mL penicillin and 100 *μ*g/mL streptomycin) for 10 days. At predetermined time intervals, 1X RIPA buffer was added to the samples to obtain the cell lysates. The* p*-nitrophenyl phosphate solution was added to the cell lysates and incubated for 30 min at 37°C. The reaction was stopped by adding 500 *μ*L of 1N NaOH. ALP activity was determined by measuring the conversion of* p*-nitrophenyl phosphate to* p*-nitrophenol. Optical density was determined using a microplate reader at a wavelength of 405 nm.

### 2.9. Calcium Deposition

We also examined the calcium depositions to determine the late osteogenic differentiation. 1 × 10^5^ cells/mL of MG-63 cells were seeded on PCL fibers and surface-modified PCL fibers into 24-well tissue-culture plates maintained in osteogenic medium (DMEM supplemented with 10% FBS, 50 *μ*g/mL ascorbic acid, 10 nM dexamethasone, and 10 mM *β*-glycerophosphate in the presence of 100 U/mL penicillin and 100 *μ*g/mL streptomycin) for 21 days. After 21 days of incubation, samples were rinsed with PBS and then 0.5N HCl was added to the samples. The samples were centrifuged at 13,500 rpm for 1 min. The calcium depositions in the resulting supernatant were measured using the QuantiChrom Calcium Assay Kit (DICA-500, BioAssay Systems, USA) according to the manufacturer's instructions. The amount of calcium produced was estimated by measuring the absorbance at 612 nm using a microplate reader.

### 2.10. Gene Expressions of Osteocalcin and Osteopontin

Osteocalcin (OCN) and osteopontin (OPN) were used to determine the effect of osteogenic differentiation on the mRNA expression levels. 1 × 10^5^ MG-63 cells were seeded onto the surface of the PCL fibers and surface-modified PCL fibers into a 24-well tissue-culture plate for 21 days. Total RNA (1 *μ*g) was used to synthesize cDNA using the Superscript First-Strand Synthesis System (Invitrogen, Carlsbad, CA, USA) according to the manufacturer's instructions. cDNA was amplified by polymerase chain reaction (PCR) using an RNA PCR kit (Bioneer Inc., Daejeon, South Korea) in accordance with the manufacturer's instructions. The primers used were OCN (F) 5′-TTG GTG CAC ACC TAG CAG AC-3′, (R) 5′-ACC TTA TTG CCC TCC TGC TT-3′; OPN (F) 5′-GAG GGC TTG GTT GTC AGC-3′, (R) 5′-CAA TTC TCA TGG TAG TGA GTT TTC C-3′; and GAPDH (F) 5′-ACT TTG TCA AGC TCA TTT CC-3′, (R) 5′-TGC AGC GAA CTT TAT TGA TG-3′. PCR amplification and detection were conducted on an ABI7300 Real-Time Thermal Cycler (Applied Biosystems, Foster City, CA, USA) using the DyNAmo SYBR Green qPCR Kit (Finnzymes, Espoo, Finland). The relative mRNA expression levels of OCN and OPN were normalized to GAPDH. Also, osteocalcin (OCN) and osteopontin (OPN) were used to determine the effect of osteogenic differentiation on the mRNA expression levels. 1 × 10^5^ MG-63 cells were seeded onto the surface of the PCL fibers and surface-modified PCL fibers into a 24-well tissue-culture plate without osteogenic medium for 21 days.

### 2.11. Mineralization Assay (Alizarin Red S Staining)

The mineralization levels were assessed by staining with Alizarin red S. 1 × 10^5^ MG-63 cells were seeded onto the surface of the PCL fibers and surface-modified PCL fibers into a 24-well tissue-culture plate for 21 days. In brief, cells were washed three times with PBS and fixed in 4% paraformaldehyde for 15 min, and 40 mmol/L of Alizarin red S was prepared in distilled water, adjusted to a pH of 4.2 with ammonium hydroxide, and then applied to the cells for 1 min at room temperature with gentle agitation. Then, samples were examined with the observer that was blinded to the group assignment under a light microscope.

### 2.12. Statistical Analysis

Quantitative data are presented as means ± standard deviation, and comparisons were carried out using one-way ANOVA (Systat Software, Inc., Chicago, IL, USA). Differences were considered statistically significant at ^∗^ *P* < 0.05.

## 3. Results

### 3.1. Characterization of PCL Fibers and Surface-Modified PCL Fibers

**Figure 1:**
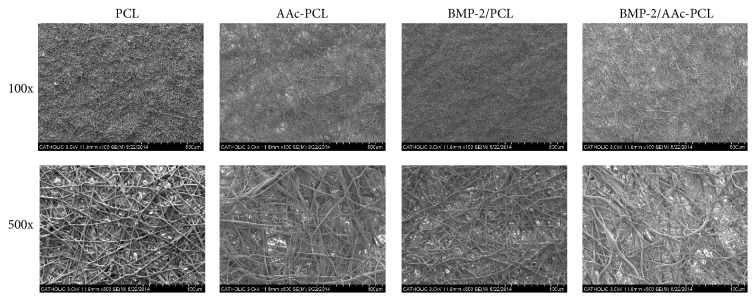
Scanning electron microscope (SEM) of PCL, AAc-PCL, BMP-2/PCL, and BMP-2/AAc-PCL.

**Figure 2:**
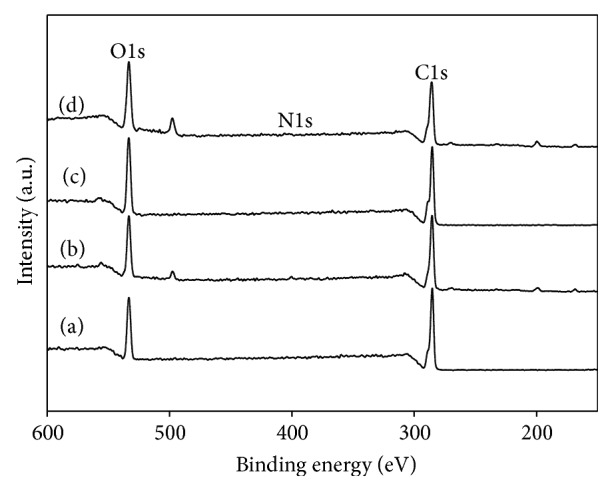
X-ray photoelectron spectroscopy (XPS) wide-scan spectra of (a) PCL, (b) AAc-PCL, (c) BMP-2/PCL, and (d) BMP-2/AAc-PCL.

PCL, AAc-PCL, BMP-2/PCL, and BMP-2/AAc-PCL fibers were evaluated using the SEM to confirm their morphologies before and after surface modification. The surface of the PCL fibers after the modification with AAc or BMP-2 showed similar morphologies to that of PCL fibers before modification (Figure 1). Moreover, the elemental chemical compositions of the PCL, AAc-PCL, BMP-2/PCL, and BMP-2/AAc-PCL fibers were performed by XPS analysis. As shown in Figure 2 and Table 1, successful AAc grafts on the surface of the PCL fibers were assessed by an increase in O1s content from 22.21% to 26.05% and a decrease in C1s content from 77.79% to 73.3%, as compared to the PCL fiber alone. In addition, the successful immobilization of BMP-2 on the surface of AAc-PCL or PCL was confirmed by an increase in N1s content from 0% to 1.28% or from 0% to 2.42%, in comparison to the PCL fibers alone. The hydrophilic properties of PCL, AAc-PCL, BMP-2/PCL, and BMP-2/AAc-PCL by the mean ± SDs of the contact angles were 103.12 ± 3.5, 61.02 ± 1.3, 37.28 ± 1.3, and 31.03 ± 0.29, respectively. The amount of grafted AAc on the surface of the PCL fibers was 0.38 mM/fiber.

### 3.2. Release Profiles of BMP-2* In Vitro*

**Figure 3:**
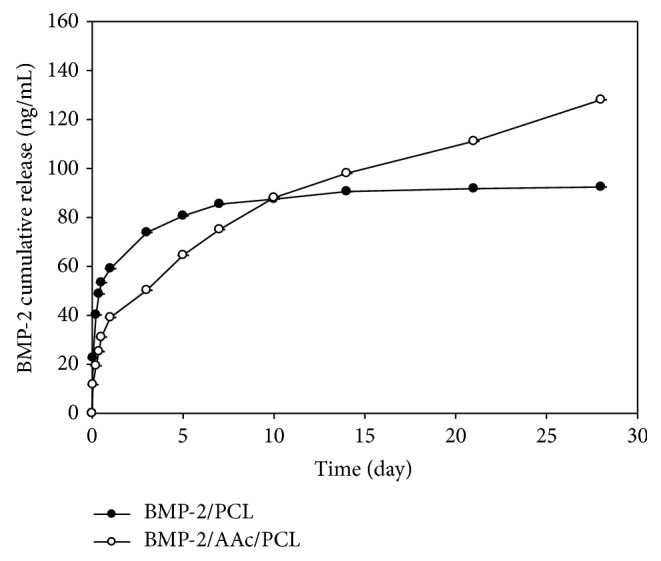
Release kinetics of BMP-2 from BMP-2/PCL and BMP-2/AAc-PCL.

The loading efficiency of BMP-2 from BMP-2/PCL and BMP-2/AAc-PCL was 77.28 and 99.16%, respectively. Also, the loading amount of BMP-2 from BMP-2/PCL and BMP-2/AAc-PCL was 386.4 and 495.8 ng, respectively. Due to the stronger electrostatic interactions, the loading efficiency of BMP-2 on AAc-PCL might have been higher than that of PCL. The release profiles of the BMP-2 from the BMP-2/PCL and BMP-2/AAc-PCL were evaluated by enzyme-linked immunosorbent assay (ELISA). As shown in Figure 3, the released amounts of BMP-2 from BMP-2/PCL and BMP-2/AAc-PCL were 57.07 ± 0.02 ng and 39.12 ± 0.03 ng at 1 day, respectively. During the study period of 28 days, the released amounts of BMP-2 were 92.47 ± 0.05 ng from BMP-2/PCL and 128.07 ± 0.04 ng from BMP-2/AAc-PCL.

### 3.3. Cell Proliferation

**Figure 4:**
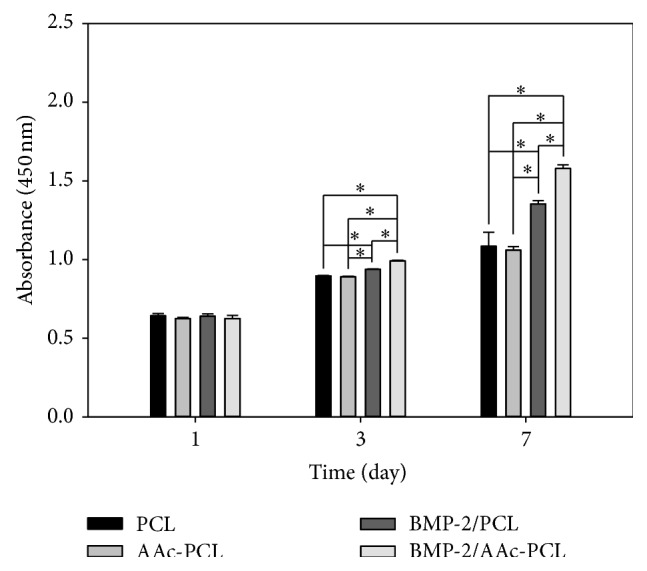
Cell proliferation of MG-63 cells grown on PCL, AAc-PCL, BMP-2/PCL, and BMP-2/AAc-PCL after 1, 3, and 7 days of incubation.

As shown in Figure 4, the cell proliferation of MG-63 cells cultured on PCL, AAc-PCL, BMP-2/PCL, and BMP-2/AAc-PCL was increased throughout the 7 days. On the 1st day, there were no significant differences in the cell proliferation of the MG-63 cells between any of the groups. There were significant differences in the cell proliferation of the MG-63 cells cultured on the BMP-2/PCL, BMP-2/AAc-PCL, and PCL on the 3rd and 7th days (^∗^ *P* < 0.05). Furthermore, on the 3rd and 7th days, significant differences were observed in the proliferation of MG-63 cells grown on the BMP-2/PCL and BMP-2/AAc-PCL (^∗^ *P* < 0.05). Furthermore, the cell proliferation of MG-63 cells grown on both BMP-2/PCL and BMP-2/AAc-PCL was significantly different (^∗^ *P* < 0.05). However, there were no significant differences in cell proliferation between the MG-63 cells cultivated on PCL and on AAc-PCL for the predetermined time intervals (^∗^ *P* < 0.05).

### 3.4. Alkaline Phosphatase (ALP) Activity

**Figure 5:**
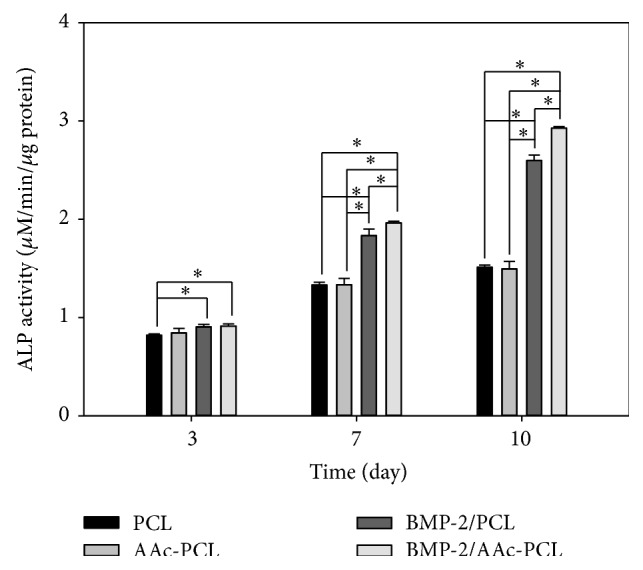
(A) Alkaline phosphatase (ALP) activity of MG-63 cells grown on PCL, AAc-PCL, BMP-2/PCL, and BMP-2/AAc-PCL after 3, 7, and 10 days of incubation.

The ALP activity of the MG-63 cells increased in all groups for the incubation period of up to 10 days (Figure 5). On the 3rd day, there were no significant differences in the ALP activity of the MG-63 cells cultivated on the AAc-PCL and PCL. However, differences in ALP activities were significant between the MG-63 cells grown on BMP-2/PCL or BMP-2/AAc-PCL and those on PCL (^∗^ *P* < 0.05). On the 7th day, the ALP activity of MG-63 cells grown on PCL containing BMP-2 increased significantly compared to the activity on PCL (^∗^ *P* < 0.05). On the 10th day, significant differences were observed in the ALP activities of MG-63 cells cultured on PCL containing BMP-2 and PCL (^∗^ *P* < 0.05). Moreover, the ALP activities were significantly different between the MG-63 cells in the BMP-2/AAc-PCL and the BMP-2/PCL on the 7th and 10th days (^∗^ *P* < 0.05).

### 3.5. Calcium Deposition

**Figure 6:**
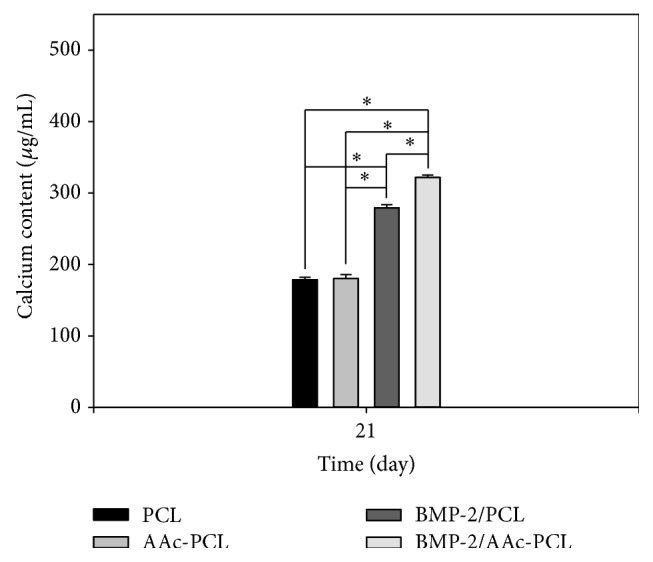
The amount of calcium deposition of the MG-63 cells grown on PCL, AAc-PCL, BMP-2/PCL, and BMP-2/AAc-PCL after 21 days of incubation.

As shown in Figure 6, the amount of calcium deposition on the MG-63 cells grown on PCL, AAc-PCL, BMP-2/PCL, and BMP-2/AAc-PCL was investigated after 21 days of culture. The amount of calcium deposited by the MG-63 cells cultivated on PCL with BMP-2 was significantly greater than that on PCL (^∗^ *P* < 0.05). Additionally, the amount of calcium deposited by MG-63 cells grown on BMP-2/AAc-PCL has markedly increased compared to that on PCL (^∗^ *P* < 0.05). However, the amount of calcium deposited by MG-63 cells was not significantly different between AAc-PCL and PCL.

### 3.6. Gene Expression

**Figure 7:**
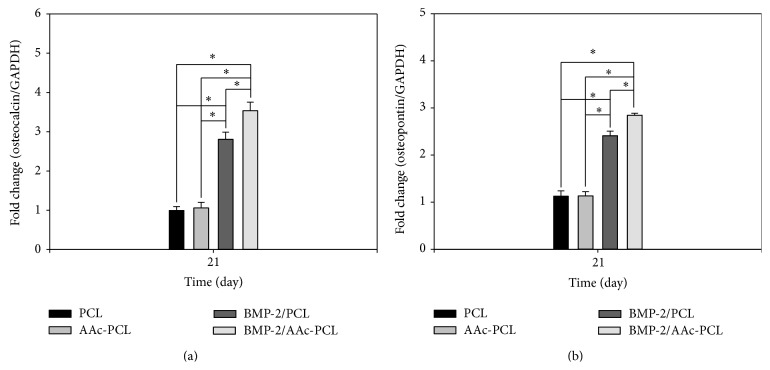
Gene expression of (a) osteocalcin and (b) osteopontin in MG-63 cells grown on PCL, AAc-PCL, BMP-2/PCL, and BMP-2/AAc-PCL after 21 days of incubation by real-time PCR analysis.

**Figure 8:**
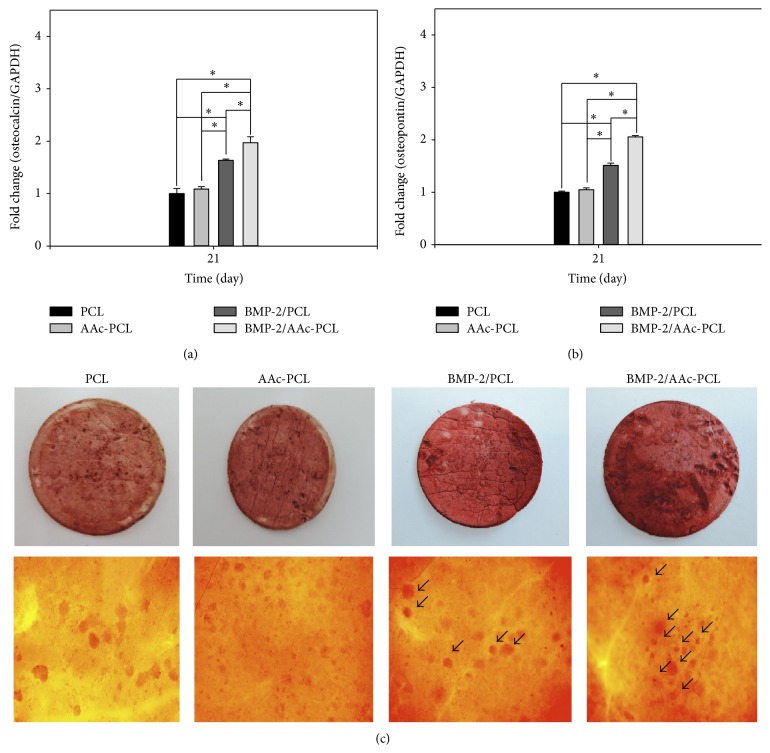
Gene expression of (a) alkaline phosphatase (ALP) and (b) osteocalcin in MG-63 cells grown on PCL, AAc-PCL, BMP-2/PCL, and BMP-2/AAc-PCL after 10 days of incubation without osteogenic medium by real-time PCR analysis. (c) Alizarin red S staining in MG-63 cells grown on PCL, AAc-PCL, BMP-2/PCL, and BMP-2/AAc-PCL after 21 days of incubation.

We investigated osteogenic differentiation on the mRNA expression levels for OCN and OPN in the MG-63 cells cultured in all groups using real-time PCR after incubation for 21 days (Figures 7(a) and 7(b)). A significant difference in OCN and OPN expression levels was observed between PCL containing BMP-2 and PCL (^∗^ *P* < 0.05). OCN and OPN expression levels in MG-63 cells grown on BMP-2/AAc-PCL were significantly greater than MG-63 cells grown on PCL (^∗^ *P* < 0.05). Meanwhile, OCN and OPN expression levels in MG-63 cells cultured on AAc-PCL and PCL were observed to be similar. We have also investigated osteogenic differentiation on the mRNA expression level for OCN and OPN in the MG-63 cells cultured without an osteogenic medium in all groups using real-time PCR, after incubation for 21 days (Figures 8(a) and 8(b)). A significant difference in OCN and OPN expression levels was observed between PCL containing BMP-2 and PCL (^∗^ *P* < 0.05). OCN and OPN expression levels in MG-63 cells grown on BMP-2/AAc-PCL were significantly greater than MG-63 cells grown on PCL (^∗^ *P* < 0.05). Meanwhile, OCN and OPN expression levels in MG-63 cells cultured on AAc-PCL and PCL were observed to be similar.

### 3.7. Mineralization Levels

We used Alizarin red S staining to examine the mineralization levels of the extracellular matrix* in vitro* when MG-63 cells were cultured in the presence of BMP-2/AAc-PCL. The formation of mineralized nodules (arrow) in the BMP-2/AAc-PCL was increased than that of the cells in the PCL and BMP-2/PCL.

## 4. Discussion

In this study, we have modified a PCL fiber using the *γ*-ray irradiation technique to graft AAc and immobilize BMP-2 on PCL fibers as well as inducing the proliferation and osteogenic differentiation of MG-63 cells. The grafted AAc on the surface of the PCL fibers using the *γ*-ray irradiation technique was confirmed by toluidine blue staining. The grafted AAc on the PCL fibers was stained blue compared with the PCL fibers. Shin et al. [19] demonstrated that the grafted AAc on the poly(L-lactide-co-*ε*-caprolactone) (PLCL) fibrous mesh using the *γ*-ray irradiation was stained with a blue color. This result indicated that PCL fibers that were stained blue were successfully anchored with AAc. In addition, the characterization of the PCL and surface-modified PCL with AAc and BMP-2 was demonstrated with XPS and contact angle measurement. Successful anchoring of the AAc onto the PCL fiber surface was displayed by an increase in O1s content and a decrease in C1s content compared to the PCL fibers. Moreover, successful coating or immobilization of BMP-2 onto the PCL or AAc-PCL fibers were demonstrated by an increase in N1s content compared to the PCL or AAc-PCL fibers. The fiber surfaces of the BMP-2 that was coated or immobilized onto the PCL or AAc-PCL fiber had a smaller contact angle than that of the PCL fiber surfaces, indicating that the former was more hydrophilic compared with the PCL fiber surface. These results indicated that BMP-2 was successfully coated or immobilized onto the surface of the PCL or AAc-PCL fiber. Kim et al. [27, 30] reported that BMP-2-immobilized heparin-grafted Ti or heparin-dopamine (Hep-DOPA)-grafted PCL fiber surfaces were more hydrophilic than pristine Ti or PCL fibers.

BMP-2 has been commercially used in orthopedic fields for spinal fusion or fracture union. Although BMP-2 has been used in various clinical treatments, the protein has a short half-life* in vivo*, a high cost, and a high dose requirement (1 mg BMP-2/mL defect) [34]. To overcome these drawbacks, previous studies have used heparin for the controlled and sustained release of BMP-2 because heparin is well known to have not only good binding affinities for various growth factors but also the ability to regulate the release of growth factors [27–30, 35–37]. In this study, we used polyacrylic acid (AAc) for the sustained release of BMP-2. In our release study, BMP-2 from BMP-2/AAc-PCL fibers in comparison to the BMP-2/PCL fibers underwent a sustained release, which may be due to the electrostatic interactions between the negative charge of AAc and the positive charge of BMP-2.

We also evaluated the PCL fibers and surface-modified PCL fibers to confirm the induction of osteoblast (MG-63 cells) activity by evaluating the cell proliferation, ALP activity, calcium deposition, and mRNA expressions of osteocalcin (OCN) and osteopontin (OPN). MG-63 cells grown on all fibers proliferated in a time-dependent manner. In addition, MG-63 cells cultured on PCL fibers or AAc-PCL fibers with BMP-2 proliferated significantly more in comparison to those on PCL fibers on the 3rd and 7th days, indicating that BMP-2 has a positive effect on MG-63 cell proliferation. This result was in agreement with previous studies conducted by Kim et al. [30] and Park et al. [38]. However, in other previous studies, there was no significant increase in cell proliferation observed in substrates with BMP-2 as compared to the substrates without BMP-2 [27–29, 39]. Thus, the effect of BMP-2 on cell proliferation is still not clear.

To demonstrate the osteogenic differentiation of MG-63 cells on PCL fibers and surface-modified PCL fibers, we assessed the ALP activity, calcium deposition, and the mRNA gene expressions of OCN and OPN. ALP activity is well known to be able to evaluate the early differentiation of osteoblast-like cells [30, 39]. The ALP activity of MG-63 cells cultured on PCL fibers and surface-modified PCL fibers was increased throughout the 10 days of culture. The ALP activity of MG-63 cells grown on PCL fibers and AAc-PCL fibers did not significantly differ on the 3rd, 7th, and 10th days. In contrast, the ALP activity of MG-63 cells grown on PCL fibers or AAc-PCL fibers with BMP-2 was significantly greater than that on PCL fibers on the 3rd, 7th, and 10th days. Moreover, the ALP activity of MG-63 cells on the BMP-2/AAc-PCL fibers surface on the 7th or 10th day was significantly higher than that on BMP-2/PCL fibers. The amount of calcium deposition was widely used to demonstrate the late differentiation of osteoblast-like cells [30, 39]. From the ALP activity data, the amount of calcium deposition on MG-63 cells cultured on AAc-PCL fibers was found to be similar compared to the PCL fibers. Meanwhile, there were no significant differences in the amount of calcium deposition on MG-63 cells cultivated on BMP-2-containing PCL fibers or AAc-PCL fibers compared to those on PCL fibers. Furthermore, significant differences in the amount of calcium deposition were shown between MG-63 cells grown on BMP-2/PCL fibers and those on BMP-2/AAc-PCL fibers. Kim et al. [27, 30] and Huh et al. [39] demonstrated that BMP-2-immobilized Ti, PCL fibers, and bovine bone substrates showed significant differences in ALP activity and calcium deposition compared to Ti, PCL fibers, and bovine bone substrates without BMP-2. As mentioned in the Results Section of these studies, BMP-2 directly stimulates the early and late differentiation of MG-63 cells.

The osteocalcin (OCN) and osteopontin (OPN) genes were typical markers of osteoblast differentiation and were also used to evaluate the upregulation of osteoblast differentiation [25, 26, 30, 40]. OCN and OPN gene expressions in MG-63 cells grown on PCL fibers or AAc-PCL fibers with BMP-2 were significantly higher than those grown in PCL fibers after 21 days of incubation. Significant differences in mRNA expression of OCN and OPN were observed between MG-63 cells cultured on BMP-2/AAc-PCL fibers and BMP-2/PCL fibers. Kim et al. [30] showed that the mRNA expression levels of OCN and OPN in periodontal ligament cells (PDLCs) cultured onto BMP-2-immobilized PCL fibers were significantly increased as compared with those in PDLCs cultured onto PCL fibers. BMP-2 can not only enhance directly ALP activity and calcium deposition but also upregulate OCN and OPN gene expression.

## 5. Conclusions

We have successfully modified the PCL fibers using the *γ*-ray irradiation technique and subsequently immobilized BMP-2 through electrostatic interactions with AAc. The BMP-2/AAc-PCL fibers showed sustained BMP-2 release over the 28-day study period in comparison to the BMP-2/PCL fibers. BMP-2/AAc-PCL fibers markedly induced the osteogenic differentiation of MG-63 cells by increasing ALP activity, calcium deposition, and OCN and OPN gene expression as compared with PCL fibers alone. In conclusion, BMP-2/AAc-PCL fibers were effective systems for regeneration in orthopedic diseases through the release of BMP-2. Further animal studies with orthopedic disease models such as the Achilles tendon rupture model and the rotator-cuff injury model are needed to investigate the effect of BMP-2/AAc-PCL fibers in clinical treatments.

## Figures and Tables

**Table 1:** Surface elemental compositions of PCL, AAc-PCL, BMP-2/PCL, and BMP-2/AAc-PCL.

	C1s (%)	N1s (%)	O1s (%)
PCL	77.79	—	22.21
AAc/PCL	73.3	0.65	26.05
BMP-2/PCL	75.13	2.42	22.45
BMP-2/AAc/PCL	71.11	1.28	27.61

## References

[1] Arrington E. D., Smith W. J., Chambers H. G., Bucknell A. L., Davino N. A. (1996). Complications of iliac crest bone graft harvesting. *Clinical Orthopaedics and Related Research*.

[2] Kneser U., Schaefer D. J., Polykandriotis E., Horch R. E. (2006). Tissue engineering of bone: the reconstructive surgeon's point of view. *Journal of Cellular and Molecular Medicine*.

[3] Zimmermann C. E., Börner B. I., Hasse A., Sieg P. (2001). Donor site morbidity after microvascular fibula transfer. *Clinical oral investigations*.

[4] Agarwal S., Wendorff J. H., Greiner A. (2008). Use of electrospinning technique for biomedical applications. *Polymer*.

[5] Glowacki J., Mizuno S. (2008). Collagen scaffolds for tissue engineering. *Biopolymers*.

[6] Khan Y., Yaszemski M. J., Mikos A. G., Laurencin C. T. (2008). Tissue engineering of bone: material and matrix considerations. *Journal of Bone and Joint Surgery—Series A*.

[7] Ashammakhi N., Wimpenny I., Nikkola L., Yang Y. (2009). Electrospinning: methods and development of biodegradable nanofibres for drug release. *Journal of Biomedical Nanotechnology*.

[8] Ghasemi-Mobarakeh L., Prabhakaran M. P., Balasubramanian P., Jin G., Valipouri A., Ramakrishna S. (2013). Advances in electrospun nanofibers for bone and cartilage regeneration. *Journal of Nanoscience and Nanotechnology*.

[9] Woo K. M., Jun J.-H., Chen V. J. (2007). Nano-fibrous scaffolding promotes osteoblast differentiation and biomineralization. *Biomaterials*.

[10] Mao J., Duan S., Song A., Cai Q., Deng X., Yang X. (2012). Macroporous and nanofibrous poly(lactide-co-glycolide)(50/50) scaffolds via phase separation combined with particle-leaching. *Materials Science and Engineering C*.

[11] Hartgerink J. D., Beniash E., Stupp S. I. (2001). Self-assembly and mineralization of peptide-amphiphile nanofibers. *Science*.

[12] Hosseinkhani H., Hosseinkhani M., Kobayashi H. (2006). Proliferation and differentiation of mesenchymal stem cells using self-assembled peptide amphiphile nanofibers. *Biomedical Materials*.

[13] Lee J.-Y., Choo J.-E., Choi Y.-S. (2009). Osteoblastic differentiation of human bone marrow stromal cells in self-assembled BMP-2 receptor-binding peptide-amphiphiles. *Biomaterials*.

[14] Dou X.-Q., Zhang D., Feng C.-L. (2013). Wettability of supramolecular nanofibers for controlled cell adhesion and proliferation. *Langmuir*.

[15] Suzuki A., Aoki K. (2008). Biodegradable poly(L-lactic acid) nanofiber prepared by a carbon dioxide laser supersonic drawing. *European Polymer Journal*.

[16] Yan D., Jones J., Yuan X. Y. (2013). Plasma treatment of electrospun PCL random nanofiber meshes (NFMs) for biological property improvement. *Journal of Biomedical Materials Research Part A*.

[17] Sankar D., Shalumon K. T., Chennazhi K. P., Menon D., Jayakumar R. (2014). Surface plasma treatment of poly(caprolactone) micro, nano, and multiscale fibrous scaffolds for enhanced osteoconductivity. *Tissue Engineering Part A*.

[18] Jaiswal D., Brown J. L. (2012). Nanofiber diameter-dependent MAPK activity in osteoblasts. *Journal of Biomedical Materials Research Part A*.

[19] Shin Y. M., Shin H., Lim Y. M. (2010). Surface modification of electrospun poly(L-lactide-co-*ε*-caprolactone) fibrous meshes with a RGD peptide for the control of adhesion, proliferation and differentiation of the preosteoblastic cells. *Macromolecular Research*.

[20] Karageorgiou V., Meinel L., Hofmann S., Malhotra A., Volloch V., Kaplan D. (2004). Bone morphogenetic protein-2 decorated silk fibroin films induce osteogenic differentiation of human bone marrow stromal cells. *Journal of Biomedical Materials Research Part A*.

[21] Liu Y., Enggist L., Kuffer A. F., Buser D., Hunziker E. B. (2007). The influence of BMP-2 and its mode of delivery on the osteoconductivity of implant surfaces during the early phase of osseointegration. *Biomaterials*.

[22] Boyne P. J. (2001). Application of bone morphogenetic proteins in the treatment of clinical oral and maxillofacial osseous defects. *Journal of Bone and Joint Surgery—Series A*.

[23] Herford A. S., Boyne P. J. (2008). Reconstruction of mandibular continuity defects with bone morphogenetic protein-2 (rhBMP-2). *Journal of Oral and Maxillofacial Surgery*.

[24] Maegawa N., Kawamura K., Hirose M., Yajima H., Takakura Y., Ohgushi H. (2007). Enhancement of osteoblastic differentiation of mesenchymal stromal cells cultured by selective combination of bone morphogenetic protein-2 (BMP-2) and fibroblast growth factor-2 (FGF-2). *Journal of Tissue Engineering and Regenerative Medicine*.

[25] Diefenderfer D. L., Osyczka A. M., Garino J. P., Leboy P. S. (2003). Regulation of BMP-induced transcription in cultured human bone marrow stromal cells. *The Journal of Bone & Joint Surgery—American Volume*.

[26] Lecanda F., Avioli L. V., Cheng S.-L. (1997). Regulation of bone matrix protein expression and induction of differentiation of human osteoblasts and human bone marrow stromal cells by bone morphogenetic protein-2. *Journal of Cellular Biochemistry*.

[27] Kim S. E., Song S.-H., Yun Y. P. (2011). The effect of immobilization of heparin and bone morphogenic protein-2 (BMP-2) to titanium surfaces on inflammation and osteoblast function. *Biomaterials*.

[28] Lee D.-W., Yun Y.-P., Park K., Kim S. E. (2012). Gentamicin and bone morphogenic protein-2 (BMP-2)-delivering heparinized-titanium implant with enhanced antibacterial activity and osteointegration. *Bone*.

[29] Lee S.-Y., Yun Y.-P., Song H.-R. (2013). The effect of titanium with heparin/BMP-2 complex for improving osteoblast activity. *Carbohydrate Polymers*.

[30] Kim S. E., Yun Y.-P., Han Y.-K. (2014). Osteogenesis induction of periodontal ligament cells onto bone morphogenic protein-2 immobilized PCL fibers. *Carbohydrate Polymers*.

[31] Alvarez-Lorenzo C., Bucio E., Burillo G., Concheiro A. (2010). Medical devices modified at the surface by *γ*-ray grafting for drug loading and delivery. *Expert Opinion on Drug Delivery*.

[32] Shim J. K., Na H. S., Lee Y. M., Huh H., Nho Y. C. (2001). Surface modification of polypropylene membranes by *γ*-ray induced graft copolymerization and their solute permeation characteristics. *Journal of Membrane Science*.

[33] Alves P., Coelho J. F. J., Haack J., Rota A., Bruinink A., Gil M. H. (2009). Surface modification and characterization of thermoplastic polyurethane. *European Polymer Journal*.

[34] Sellers R. S., Zhang R., Glasson S. S. (2000). Repair of articular cartilage defects one year after treatment with recombinant human bone morphogenetic protein-2 (rhBMP-2). *The Journal of Bone and Joint Surgery—American Volume*.

[35] Ishibe T., Goto T., Kodama T., Miyazaki T., Kobayashi S., Takahashi T. (2009). Bone formation on apatite-coated titanium with incorporated BMP-2/heparin in vivo. *Oral Surgery, Oral Medicine, Oral Pathology, Oral Radiology and Endodontology*.

[36] Park J. S., Park K., Woo D. G., Yang H. N., Chung H.-M., Park K.-H. (2008). Triple constructs consisting of nanoparticles and microspheres for bone-marrow-derived stromal-cell-delivery microscaffolds. *Small*.

[37] Sasisekharan R., Ernst S., Venkataraman G. (1997). On the regulation of fibroblast growth factor activity by heparin-like glycosaminoglycans. *Angiogenesis*.

[38] Park Y. J., Kim K. H., Lee J. Y. (2006). Immobilization of bone morphogenetic protein-2 on a nanofibrous chitosan membrane for enhanced guided bone regeneration. *Biotechnology and Applied Biochemistry*.

[39] Huh J.-B., Kim S.-E., Song S.-K. (2011). The effect of immobilization of heparin and bone morphogenic protein-2 to bovine bone substitute on osteoblast-like cell's function. *Journal of Advanced Prosthodontics*.

[40] Yun Y.-P., Kim S. E., Kang E. Y., Kim H.-J., Park K., Song H.-R. (2013). The effect of bone morphogenic protein-2 (BMP-2)-immobilizing heparinized-chitosan scaffolds for enhanced osteoblast activity. *Tissue Engineering and Regenerative Medicine*.

